# Radiologic Imaging in Third Nerve Palsy: A Case Series Investigating Etiology, Patterns, and Clinical Implications

**DOI:** 10.7759/cureus.43986

**Published:** 2023-08-23

**Authors:** Surbhi A Chodvadiya, Varsha Manade, Megha Kotecha, Jhimli Ta

**Affiliations:** 1 Department of Ophthalmology, Dr. D.Y Patil Medical College, Hospital and Research Centre, Pune, IND

**Keywords:** oculomotor nerve, diplopia, ptosis, imaging, third nerve palsy

## Abstract

Third nerve palsy (TNP) is a neurologic condition characterized by dysfunction of the oculomotor nerve, leading to various ocular manifestations. Optic nerve evaluation is of utmost important among all cranial nerve palsies affecting the eye. Dysfunction of the third nerve can indicate an underlying neurologic emergency, such as cavernous arteriovenous fistula or giant cell arteritis. Early recognition and prompt treatment are vital in reversing the clinical and visual impairments associated with oculomotor nerve palsy. The typical presentation of isolated TNP involves deviation of the eye in a downward and outward direction, accompanied by ptosis (drooping of the eyelid) and, potentially, pupil involvement. The decision to use vascular imaging is influenced by factors such as age and clinical risk for an aneurysm. If TNP is isolated or partially present with pupil involvement, it suggests compression of the third nerve and necessitates immediate imaging. Given the serious implications of an intracranial aneurysm, physicians often prioritize vascular imaging during the initial evaluation, if available. However, if clinical findings indicate underlying microvascular ischemia, a delay in imaging may be considered. This case series aims to explore the role of radiologic imaging in understanding the etiology, patterns, and clinical implications of TNP.

## Introduction

Third nerve palsy (TNP), also known as oculomotor nerve palsy, refers to a neurological condition characterized by dysfunction or damage to the third cranial nerve (oculomotor nerve) [1]. The oculomotor nerve is responsible for controlling the movement and function of several eye muscles, including those responsible for eye movement, pupil constriction, and eyelid elevation. It supplies motor fibers to the extraocular muscles of the globe and levator muscle of the eyelid and parasympathetic pupillomotor fibers to the ciliary ganglion [2]. When the third cranial nerve is affected, it can result in various symptoms and visual disturbances. These may include eye movement abnormalities: TNP can lead to limited or complete loss of voluntary eye movements, particularly affecting the ability to move the affected eye inward, upward, and downward. It can also lead to ptosis (drooping of the eyelid on the affected side due to the involvement of the muscle responsible for eyelid elevation, that is the levator palpebrae superioris muscle), diplopia (double vision can occur due to the misalignment of the eyes resulting from the imbalance of the affected eye muscles such as the superior rectus, inferior rectus, medial rectus, and inferior oblique muscle), pupil abnormalities (the affected eye may exhibit a dilated pupil that does not constrict properly in response to light), and eye misalignment (the affected eye may deviate outward and downward due to lack of action of the muscles controlled by the third cranial nerve, such as superior rectus, inferior rectus, medial rectus, and inferior oblique muscle, and unopposed action of the lateral rectus muscle and superior oblique muscle, which are supplied by the abducent nerve and trochlear nerve, respectively).

Written well-informed patients' consent was taken for this case series.

## Case presentation

Case 1 (trauma)

**Figure 1:**
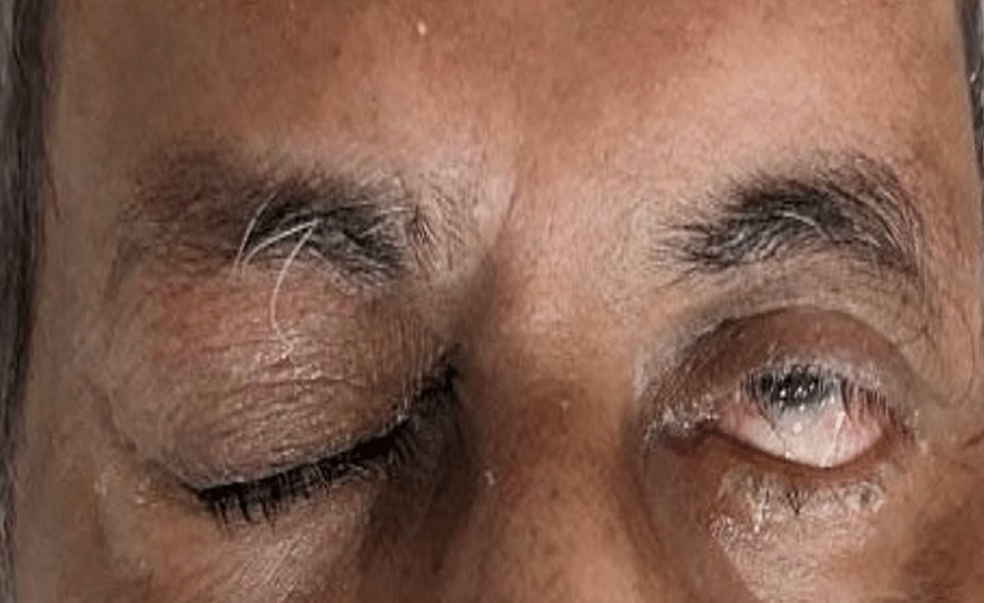
Ptosis in the right eye

**Figure 2:**
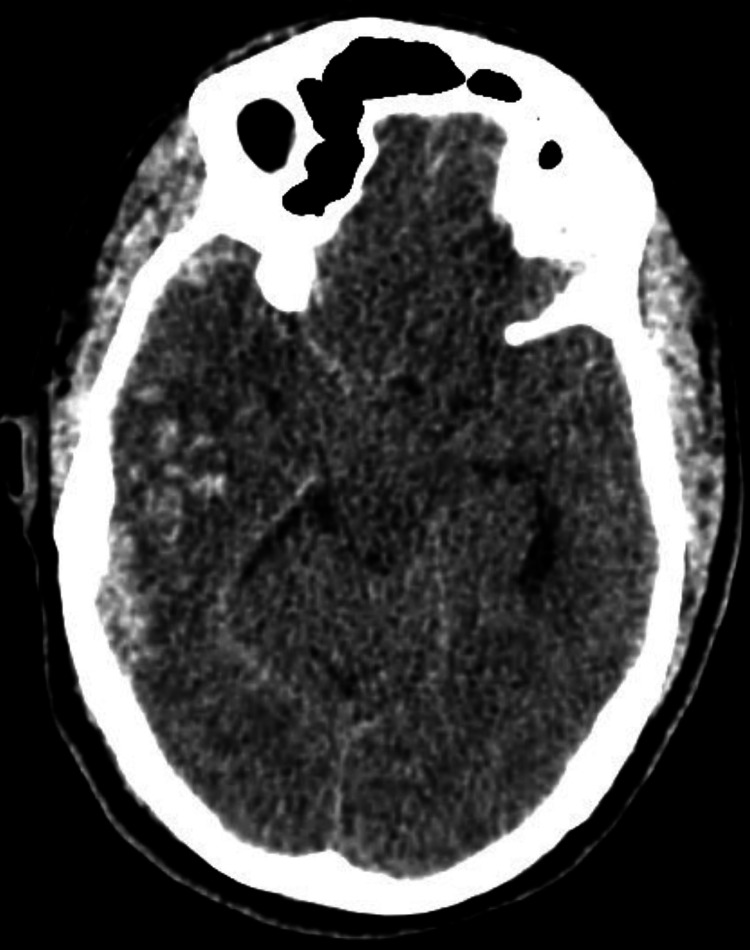
Subarachnoid hemorrhage in the right temporal region

**Figure 3:**
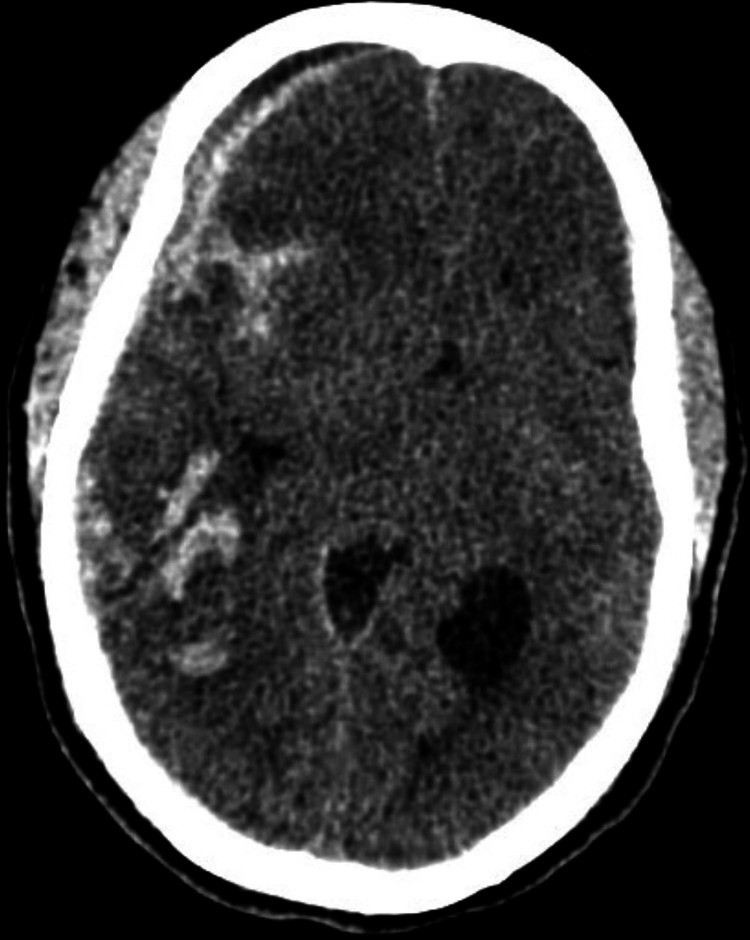
Subarachnoid hemorrhage in the right frontal region

A 53-year-old man was brought to the emergency department (ED) following a motorbike accident involving a collision with a car. A bystander discovered him unconscious while still wearing his helmet. While en route to the hospital, he regained consciousness but experienced retrograde amnesia. We received a referral for an ophthalmic evaluation. During the examination, he was fully conscious, he had complete ptosis (drooping of the eyelid) in his right eye (Figure 1), and his right eye had a visual acuity of 6/9. We observed complete ptosis with an outward deviation of the eye (exotropia). Pupillary examination revealed a sluggish, dilated pupil in the right eye measuring 7 mm, with no reverse relative afferent pupillary defect. The patient exhibited limited eye movement in upward, downward, and inward directions, leading to double vision (diplopia). All other cranial nerves confined to the eyeball, such as the abducent nerve, and trochlear nerve functions were intact. A computed tomography (CT) scan of the brain and orbit showed an acute subarachnoid hemorrhage in the right temporal region (Figure 2) and the right frontal region (Figure 3). No evidence of fractures in the orbital wall or entrapment of the extraocular muscles was observed. The patient remained stable during the initial 24-hour monitoring period and was diagnosed with right eye pupil-sparing complete TNP with traumatic mydriasis. A follow-up CT scan of the brain conducted five days later revealed a complete resolution of the subarachnoid hemorrhage. The patient's clinical condition improved after two weeks, with the resolution of ptosis and recovery of ocular motility. Of note, trauma accounts for approximately 12% of all TNP cases [3].

Case 2 (thrombosis of the superior sagittal sinus, inferior sagittal sinus, right transverse sinus, and right sigmoid sinus)

A 47-year-old man with newly diagnosed hypertension presented to the ED with an eight-day history of double vision associated with headache (frontal and occipital) for five days that was sharp in nature, vomiting, and neck pain. He had no other neurologic symptoms. His blood pressure (BP) was 199/88 mm Hg. Once his BP was stabilized, he was referred to us for a detailed eye assessment. His best corrected visual acuity was 6/12 bilaterally. Upon examination, it was observed that his left eye deviated outward and downward, resulting in a misalignment of the eyes. This misalignment caused the patient to see two distinct images instead of a single, unified image, and it is termed “diplopia.”

Further assessment of the patient's eye movements revealed limited ability to move the left eye upward, downward, and inward. There was no proptosis, and intraocular pressure was normal. Fundus examination of the left eye was unremarkable. All other cranial nerve and neurologic examinations were normal. We diagnosed left eye pupil-involving TNP.

**Figure 4:**
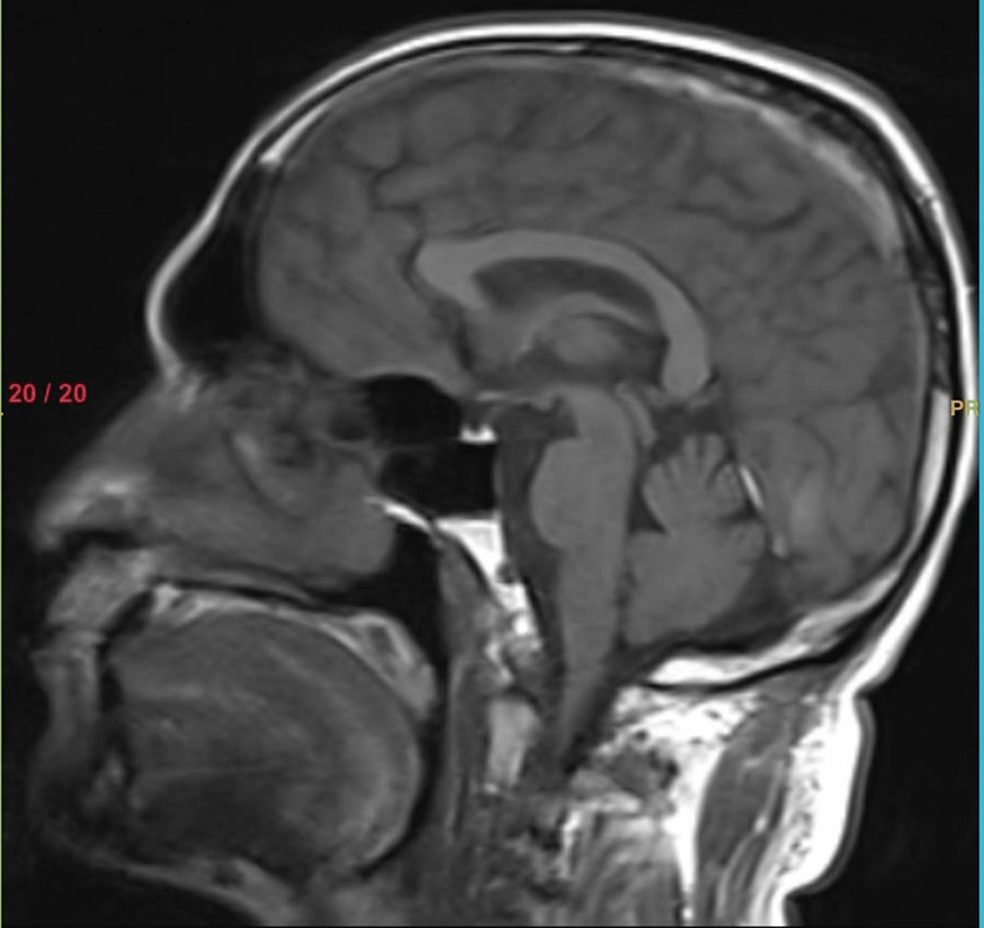
Thrombosis of the superior sagittal sinus, inferior sagittal sinus, right transverse sinus, and right sigmoid sinus

Further assessment of the brain with magnetic resonance imaging (MRI) revealed thrombosis of the superior sagittal sinus, inferior sagittal sinus, right transverse sinus, and right sigmoid sinus (Figure 4). Treatment for the patient involved addressing the cerebral venous sinus thrombosis, which typically includes the use of anticoagulant medications to prevent further clot formation and promote blood flow restoration. Close monitoring of the patient's condition and regular follow-up examinations were initiated to assess the resolution of symptoms and any potential complications.

Case 3 (splenium of corpus callosum infarct)

**Figure 5:**
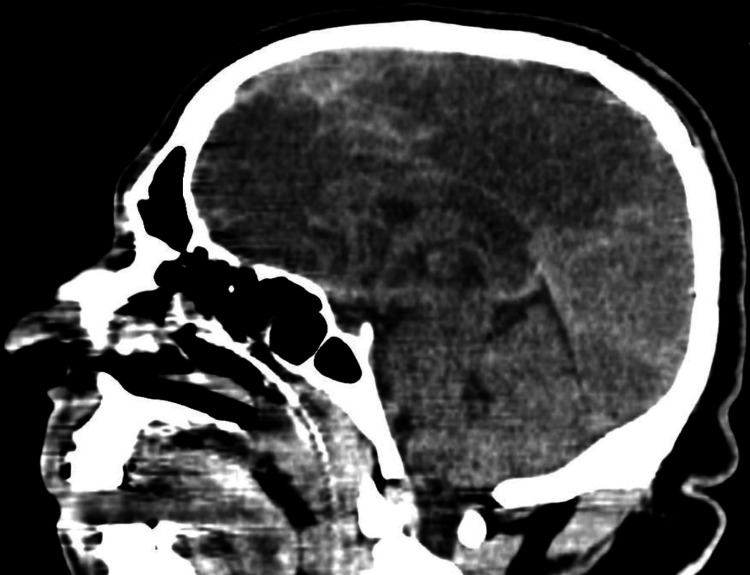
Splenium of corpus callosum infarct

A 60-year-old man presented to the neurology department with a complaint of imbalance while walking and a history of two falls in one month and was referred to us for restricted upward gaze, gaze-evoked nystagmus, and double vision. Upon examination, it was observed that the patient's right eye displayed ptosis. This ptosis resulted in a partially obstructed visual field for the affected eye. In addition, the right eye exhibited limited movement in various directions, including upward and inward gaze. The patient also experienced a noticeable deviation of the right eye, causing misalignment with the left eye. Consequently, he reported experiencing double vision (diplopia) due to the misalignment of the eyes. To establish the diagnosis and determine the underlying cause, the patient underwent various diagnostic procedures, including a thorough neurological examination and neuroimaging studies such as CT scan (Figure 5). The imaging confirmed the presence of a splendid corpus callosum infarct, providing insight into the cause of the TNP, and both the optic tracts appeared to be intact on the CT scan.

These ocular manifestations are attributed to the splendid corpus callosum infarct. The infarct disrupted the communication between the brain hemispheres, particularly affecting the fibers related to the third cranial nerve. The third cranial nerve controls the muscles responsible for eye movements, pupil constriction, and eyelid elevation. The disruption of this nerve's function resulted in the observed ptosis, limited eye movements, and misalignment of the right eye.

## Discussion

The presented case series explores the role of radiologic imaging in understanding the etiology, patterns, and clinical implications of TNP. The discussion will focus on the findings of each case and their significance in enhancing our understanding of this neurologic condition.

In case 1, the patient experienced TNP following a traumatic motorbike accident. The presence of complete ptosis, exotropia, and limited eye movements indicated the involvement of the third cranial nerve. Radiologic imaging in the form of a CT scan revealed an acute subarachnoid hemorrhage in the right frontotemporal region [4]. This finding highlights the importance of considering traumatic etiologies in cases of TNP and the need for prompt imaging to identify any associated intracranial pathologies. It has been suggested that TNP is more frequently observed in cases of frontal region injury [5].

Case 2 presents a patient with TNP caused by thrombosis of multiple sinuses. The presence of double vision [6], limited eye movements, and misalignment of the eyes prompted further investigation. MRI revealed thrombosis in the superior sagittal, inferior sagittal, right transverse, and right sigmoid sinuses. This case underscores the significance of considering vascular etiologies, such as cerebral venous sinus thrombosis, in patients presenting with TNP and emphasizes the role of radiologic imaging in diagnosing these conditions.

In case 3, the patient exhibited TNP symptoms resulting from a splendid corpus callosum infarct. The presence of ptosis, limited eye movements, and eye misalignment suggested the involvement of the third cranial nerve. Radiologic imaging in the form of a CT scan confirmed the presence of the corpus callosum infarct. This case highlights the importance of considering central nervous system lesions as potential causes of TNP and demonstrates the value of radiologic imaging in identifying these underlying pathologies.

Collectively, these cases demonstrate the diverse etiologies associated with TNP and emphasize the importance of radiologic imaging in identifying these causes. The findings contribute to our understanding of the underlying mechanisms and patterns observed in TNP [7]. The ability to identify specific etiologies through radiologic imaging enables clinicians to tailor treatment approaches accordingly.

The discussion also addresses the clinical implications of the study findings. Early recognition and prompt treatment are crucial in cases of TNP, as they can signal underlying neurologic emergencies and potential life-threatening conditions such as cerebral aneurysms or thrombosis [8,9]. Radiologic imaging plays a vital role in guiding treatment decisions, facilitating timely interventions, and improving patient outcomes [10].

In cases of congenital TNP, radiologic imaging, such as MRI or CT scans, can play a crucial role in assessing the underlying anatomical abnormalities that might be causing the condition [11]. Imaging can help identify structural anomalies, such as issues with the brainstem, nerves, or surrounding structures, which could be affecting the development and function of the third cranial nerve [12]. Radiologic imaging can provide valuable information to guide treatment decisions and management plans. If you suspect congenital TNP, consulting with a medical professional, preferably a pediatric neurologist or ophthalmologist, is essential to determine the appropriate diagnostic approach.

The retrospective nature of the study and the limited number of cases may introduce selection bias and impact the generalizability of the findings. Additionally, further research is needed to validate and expand upon these findings, including larger prospective studies and the exploration of advanced imaging techniques [13].

## Conclusions

This case series sheds light on the significance of radiologic imaging in understanding the etiology, patterns, and clinical implications of TNP. The findings highlight the importance of considering various etiologies, such as trauma, thrombosis, and central nervous system lesions, in patients presenting with TNP. Radiologic imaging provides valuable insights into these underlying causes, guiding appropriate treatment strategies and improving patient care. Further research is warranted to enhance our understanding of this condition and optimize diagnostic and therapeutic approaches.
